# Flu vaccination among patients with diabetes: motives, perceptions, trust, and risk culture - a qualitative survey

**DOI:** 10.1186/s12889-018-5441-6

**Published:** 2018-05-02

**Authors:** Pierre Verger, Aurélie Bocquier, Chantal Vergélys, Jeremy Ward, Patrick Peretti-Watel

**Affiliations:** 1 Aix Marseille Univ, IRD, AP-HM, SSA, VITROME, IHU-Méditerranée Infection, Marseille, France; 2 ORS PACA, Southeastern Health Regional Observatory, Faculté de médecine, 27, Boulevard Jean Moulin, 13385 Marseille Cedex 5, France; 3 INSERM, F-CRIN, Innovative Clinical Research Network in Vaccinology (I-REIVAC), GH Cochin Broca Hôtel Dieu, Paris, France; 4 Université Paris Diderot, UMR 8236 (LIED), Paris, France

**Keywords:** Influenza vaccines; diabetes mellitus, Attitude to health, Qualitative research

## Abstract

**Background:**

Vaccination against seasonal influenza (SIV) is recommended for patients with diabetes, but their vaccination coverage is unsatisfactory in France and elsewhere. This qualitative survey of people with diabetes sought to explore 1) the extent to which SIV-related behaviour is more or less automatic; 2) reasons they choose/reject SIV; 3) their trust/distrust in authorities, science, and medicine.

**Methods:**

We conducted semi-structured in-depth interviews of 19 adults with diabetes in 2014. We recruited them through physicians or patient associations and implemented an analysis of thematic content.

**Results:**

Eight patients were vaccinated against flu in the preceding flu season and 11 were not. SIV uptake and refusal were stable over time and justified by multiple arguments. Coupons for free vaccines and regular doctor visits contributed to the habit of vaccination. Vaccination decisions were frequently anchored in past experiences of influenza and its vaccine. Patients often justified non-vaccination with attitudes of trivialisation/relativisation of influenza-associated risks and the perception that these can be controlled by means other than vaccination (e.g., through the avoidance of exposure). Some misbeliefs (e.g., SIV causes influenza) and doubts about SIV effectiveness and safety also existed. Several patients reported increased mistrust of SIV since the A/H1N1 pandemic in 2009. Patients trusted their doctors strongly regardless of their SIV behaviour, but unvaccinated patients had little trust in the government and pharmaceutical companies. Some discordances were found between perceptions and behaviour (e.g., remaining vaccinated despite doubts about SIV effectiveness or remaining unvaccinated despite feelings of vulnerability towards influenza complication), suggesting the existence of some vaccine hesitancy among patients.

**Conclusion:**

This study among patients with diabetes suggest that SIV uptake is stable, thanks to a favourable environment. Nonetheless, SIV refusal is also stable over time. Unvaccinated patients used multiple arguments to justify SIV refusal, including compensatory health beliefs. Physicians should take every opportunity to recommend SIV. The necessary individualised patient education regarding SIV requires better physician training in patients priorities. While almost all patients strongly trust their doctors, unvaccinated patients distrust distal stakeholders: it is absolutely essential to restore trust in them and to develop new more effective influenza vaccines.

## Background

Because diabetes is associated with a substantial increase in morbidity and mortality risks associated with seasonal influenza [1, 2], vaccination against it (SIV) is recommended for people with diabetes [2–4]. Nonetheless, vaccination coverage among this population does not reach the international — and French — goal of 75% [5, 6]. Prior research indicates that SIV uptake and refusals are each consistent over time [6, 7]. Most quantitative and qualitative studies of the reasons for these behaviours among the general population have referred to the Health Belief Model (HBM) hypotheses [8]. Their results suggest that SIV uptake depends on perceived severity of seasonal influenza, vulnerability to it, and the risks and effectiveness of SIV [9]. This effectiveness for the oldest patients and those with chronic diseases remains debated among scientists [10, 11]. Negative opinions about SIV were revived during the pandemic A/H1N1 mass vaccination campaign, in France especially [12]. Far fewer studies have examined the factors associated with SIV uptake among people with chronic conditions such as diabetes than among the general population. Consistent with findings among the latter, they report that SIV is less accepted by young adults, people with a low educational level, or who lack awareness for the need of SIV, or who trivialise influenza; self-perceived vulnerability to influenza has been associated with poor self-perceived health, a severe condition, and multimorbidity [6, 13–16]. Today, as vaccination reluctance expands [17, 18], doctors’ recommendations remain a key driver of SIV uptake [9, 15, 19]. Several authors have underscored the role of multiple forms of distrust as a general societal dimension essential for understanding contemporary decisions about vaccination [20, 21]. Disillusionment with science in general, defined as the turning of scientific scepticism against science itself [22], may influence trust in information received about vaccine effectiveness and safety. Several public health controversies (A/H1N1 pandemic, Mediator®, etc.) have called into question some decisions by health authorities and pointed out conflicts of interests among experts [23, 24].

We conducted a qualitative survey in southern France in 2014 among people with diabetes to study 1) the extent to which SIV uptake is more or less automatic; 2) the reasons for choosing or rejecting SIV; 3) and their trust/distrust towards authorities, science and medicine.

## Methods

### Population

We built a convenience sample of adult patients (18 years or older) with diabetes (types 1 or 2), vaccinated or not against seasonal flu during the last flu season, residing in the south of France. Participants were recruited through physicians or through patient associations at various sites of consultation or hospitalisation (Table 1). The study was presented to potential participants to obtain their agreement to participate and to make an appointment for the interview. They provided written consent at the interview.

**Table 1:** Characteristics of the sample (qualitative study of patients with diabetes)

Nb	Age (years)	Sex	Education level	Recruitment place	Length of interview (min)^a^	DM^b^type	Years since DM^b^diagnosis	SIV^c^
01	50–64	M	> Graduated high school	Patients’ association	40	1	15+	no
02	18–34	F	> Graduated high school	Patients’ association	24	1	15+	no
03	50–64	F	Graduated high school	Hospitalised in the endocrinology department	54	1	15+	no
04	65+	M	< Graduated high school	Hospitalised in the endocrinology department	29	2	10–14	no
05	50–64	F	< Graduated high school	Hospitalised in the endocrinology department	22	2	15+	yes
06	50–64	F	> Graduated high school	Hospitalised in the endocrinology department	17	1	5–9	no
07	65+	M	< Graduated high school	Endocrinology consultation	13	2	15+	yes
08	65+	F	< Graduated high school	Endocrinology consultation	12	1	15+	yes
09	65+	M	< Graduated high school	Hospitalised in the endocrinology department	48	1	15+	yes
10	35–49	M	> Graduated high school	Insulin pump consultation	39	1	15+	no
11	18–34	F	Graduated high school	Insulin pump consultation	14	1	10–14	yes
12	65+	F	> Graduated high school	Primary-care consultation	39	2	10–14	yes
13	65+	M	< Graduated high school	Primary-care consultation	24	2	5–9	yes
14	65+	M	> Graduated high school	Primary-care consultation	29	2	< 5	no
15	50–64	M	< Graduated high school	Patient association	46	1	15+	no
16	65+	F	Graduated high school	Hospitalised in the endocrinology department	35	2	15+	no
17	50–64	F	< Graduated high school	Primary-care consultation	36	2	5–9	no
18	35–49	F	< Graduated high school	Hospitalised in the endocrinology department	23	1	15+	yes
19	50–64	M	> Graduated high school	Primary-care consultation	39	2	< 5	no

^a^Some patients had less to say, hesitated less often, or went to the point more directly, while others commented profusely, or needed time to think about their responses, or digressed

^b^Diabetes mellitus

^c^Seasonal influenza vaccine for the 2013–2014 season

### Instrument and procedure

Between May to September, 2014, a sociologist (CV) conducted in-depth face-to-face interviews using a semi-structured interview guide, drafted by two sociologists and two epidemiologists. This guide addressed patients’ disease history, their relationships with and confidence in their physicians, their sources of SIV information, their SIV history, the motives of their decision about SIV, and their perceptions and beliefs about influenza, vaccination in general, and SIV (Appendix 1). All interviews were audiotaped with the participants’ consent and later transcribed verbatim. After their interviews, participants also completed a short questionnaire about their age category, sex, educational level, and the type and age at onset of their diabetes. Recruitment of new patients stopped when the preliminary thematic analysis showed data saturation.

### Data analysis

Analysis of thematic content. Two researchers (CV and PV) analysed each subject’s discourse according to its thematic content [25], applying this three-step method to each transcript: (1) an initial coding to identify emerging themes; (2) labelling of conceptual themes to establish a grid and rubrics for the thematic analysis; and (3) in-depth analysis of all transcripts to study the internal logic of each discourse, the variability of influenza- and SIV-related perceptions [8], and the types of reasons for accepting or rejecting the vaccination. At each stage, the two researchers compared the results of their separate analyses and resolved divergences by discussion.

## Results

We included 19 participants (Table 1): 8 vaccinated in the preceding flu season and 11 not.

### Regularity of SIV behaviour

All but one vaccinated patient had had SIV regularly for the past several years, without hesitation, almost automatically:

“... *When I was a kid my parents made me do it and since I’ve been an adult I do it too*” (P11).

Several reported that they had followed their physicians’ recommendations.

“…*we talked about it,* [the GP] *explained it to me at the time, I don’t remember them anymore, she said to me, it’s to your advantage, so I said, yes, ok*” (P9).

The majority reported that they had used the coupons for vaccines that they received annually from their national health insurance fund to obtain the vaccine free of charge.

Among the unvaccinated patients, the majority had never had a SIV. Two patients reported that they had not been informed about SIV and were not interested. The other learned about it either by receiving the free vaccine coupons or through their physician’s recommendation or both.

*“I receive* [the coupon for] *the vaccination every year free, as does my son, because he also has diabetes, and no, we refuse ...”* (P3)*.*

For one patient, the physician, a homeopath, advised the patient against it. Several patients reported that their physicians had never mentioned SIV and that they had not taken the initiative to talk to the doctors about it, even after receiving the coupon. This suggests that the doctors missed opportunities to convince their patients:

*“I am concerned about it, but I didn’t do it… I have been getting it* [the coupon] *for two years and I didn’t do anything, and well, I will only do it if she* [the GP] *really tells me to”* (P14).

### Perceived vulnerability and perceptions of influenza severity

Most vaccinated patients mentioned their vulnerability to influenza because of their health status and perceived that influenza can be serious, especially when they had experienced it (Table 2).

“*…with diabetes I was in danger several times… I had very severe influenza … I had to be hospitalised… I was really afraid”* (P11)*.*

But several did not feel that their diabetes made them especially vulnerable in general.

*“... I feel good, I live with it! Listen, I ride a bike, I’m in a hiking club so I walk, and I never have problems because well I might have hyperglycaemia or hypoglycaemia or whatever, but I never have this kind of problem”* (P13)*.*

Few of the unvaccinated participants also knew the risks of influenza for people with diabetes (one because he had experienced it). Nonetheless, most downplayed influenza by diverse arguments (Table 3): not a priority compared with diabetes; not serious compared with other more contagious infectious diseases; had never had it; didn’t need to be vaccinated.

*“Young patients they already mostly don’t take enough care of their diabetes ... how do you want them to put themselves out about one more vaccine?*” (P1)*.*

*“Influenza, it’s, it’s just an instant, oh, I have the flu it’s ok, in 3 days I’m better....”* (P1).

Two patients displayed fatalistic attitudes about the risks related to their diabetes.

*…finally I only treat* [the flu] *if I have it... –*

Interviewer: Knowing that it will decontrol your diabetes? –

*“Yes... is there anything that doesn’t?”* (P2)*.*

Most did not feel vulnerable to seasonal influenza because they were young or had not had complications or felt that their diabetes was well controlled. Half the unvaccinated patients considered that the risk of influenza could be controlled, through the avoidance of exposure or the availability of treatment (Table 3) or, for one patient, because he felt “mentally and physically” able to resist it.

*“I think that being a strong person, mentally and physically, I can still fight ... against the flu”* (P3)*.*

**Table 2:** Perceptions of influenza and its vaccine: vaccinated patients with diabetes (*N* = 8)

Category	n	*Examples from interview transcript*s
Feeling of vulnerability to influenza	5	*I know that the flu in a person with diabetes, it can have serious consequences...* (P12)*.*
No feeling of vulnerability due to diabetes	3	*... I feel good, I live with it! Listen, I ride a bike, I’m in a hiking club so I walk, and I never have problems because well I might have hyperglycemia or hypoglycemia or whatever, but I never have this kind of problem* (P13).
Influenza: disease perceived or experienced as serious	7	*... I know that the flu in people with diabetes can have serious consequences...* (P12)*.* *Because I’ve been sick: I’ve had the flu…* (P11).
The vaccine protects	8	*Because I told myself that in any case it’s better to have a little vaccination than to have 40 ° C of fever, because that* [the influenza vaccine] *cuts it back some* (P18).
The vaccine protects others	1	*So I say that... getting vaccinated protects yourself and protects your children but it also protects other people because otherwise it can spread...* (P12).
The vaccine is not always effective	1	*I have always done it even though some years I got the shot and then I was sick anyway; so sometimes I was tempted to stop the next year.* (P13).
The vaccine can have side effects, but not serious or much less important than its advantages	3	*... I know that it can induce muscle aches or things like that, but, well...* (P13). *... I have never considered not doing it because the risks you have from a vaccine are minor compared with what they spare us...* (P11).
Adjuvants		
Perception of low risk	3	*... I know there are aluminum salts but it is infinitesimal compared with.... we eat aluminum salts, they’re in our food...* (P12).
Admission of ignorance and fatalism	1	*... well, I know that there are some (aluminum salts) but I don’t know what they are, exactly; in any case, there are things in all drugs...* (P13).

**Table 3:** Perceptions of influenza and its vaccine: unvaccinated patients with diabetes (*N* = 11)

Category	n	*Examples from interview transcript*s
Perception that influenza is serious	2	*... I know that flu that’s not treated can get very bad…* (P1).
Trivialisation of influenza	9	*Is it really useful to protect yourself from influenza... I think you get it more or less every year…* (P19).
No feeling of vulnerability	8	*Then there was ... I ... except for the diabetes I’m rarely ill, which is why I never go to the GP, by the way; it’s a disease certainly, but one that is not at all constraining once you know how to handle it well, and as a result I absolutely do not feel ill and so I do nothing in particular to take care of my health* (P10).
General feeling of vulnerability	2	*This process of early aging of the arteries, vessels, of the organism. I am in a body, inside a person 80 years old and you know, it’s hard* (P3).
Attitudes of fatalism, relativisation	2	*Well, if I have to catch* [the flu]*, I will* (P3). *The really contagious diseases, ah! Yes, there I say yes ok* [to vaccination]*! [...] but really, the flu, no!...* (P3).
Influenza is a manageable risk	6	*I think especially that I try to pay attention to not expose myself, um... although the flu is a virus I think, no?* (P14)*.* *...after all we have treatments that can combat the flu, I mean, it’s not 20 or 50 years ago* (P3).
The vaccine is not always effective	6	*Because I had a friend who did it and that did not stop her from having a violent case of the flu* (P17).
Vaccination amounts to injecting yourself with the disease	2	*I have never had the flu, I am not going to catch it by being vaccinated...* (P17).
Invocation of innate immunity	2	*And then, I figure that the body must have what’s required to pull through by itself...* (P6).
Mistrust of side effects	3	*For example, what I heard about the people who have multiple sclerosis after hepatitis vaccinations... well, I knew several people with multiple sclerosis... Finally it is maybe not linked, because I think that nothing has been proven, but I still have this doubt!* (P6). *Pay attention to these side effects... because you know everything that is medication, now vaccines, it’s the same, you understand that there are side effects that you have to watch out for!* (P16).
Suspicion of the vaccine because of the pharmaceutical industry’s financial interests and since the H1N1 campaign	4	*… I say to myself that they monitor them ... finally they don’t do enough tests to make sure about side effects* (P10). *...I think that came from the last vaccination campaign... it was really a bloody fiasco... we had the impression that they vaccinated people for... actually we don’t know why!* (P19).
Negligence, procrastination	3	*So I should have redone the vaccine, I didn’t have any block against it in principle that... I didn’t have time, I don’t know...you know, getting through the day is not always simple* (P15).

### Perceptions related to SIV

Among those vaccinated, only one thought that the vaccine is not always effective because he was ill several times after SIV and was thus tempted to stop it (P13, Table 2). Another attributed to the SIV a greater effect than it really has.

*“I take it* [the vaccine]*... I go to the doctor and it’s done and I have no more cold, no nothing…”* (P9)*.*

Only one patient spontaneously invoked herd immunity.

“…*in getting vaccinated, we protect ourselves and we protect our children, but we also protect others, because otherwise, it spreads*” (P12).

Vaccinated patients did not perceive the vaccine as dangerous: side effects were considered banal. Several patients had heard about controversies concerning the presence of aluminium in some vaccines, but two considered the risks minimal, one trusted the SIV, and another displayed a fatalistic attitude (Table 2).

Half of the unvaccinated people reported that SIV is not (always) effective: they reported a personal experience of a severe case of influenza after SIV, or such an experience for family members or friends.

*“…the only two times that I was vaccinated against flu, I had it cataclysmically both times, really cataclysmically!”* (P1)*.*

Several patients raised questions about vaccine effectiveness because of the evolution of viral strains; others reported that vaccination amounted to injecting themselves with the disease. Some unvaccinated participants reported that it seemed preferable to them to be immunised naturally rather than to be vaccinated. Several feared serious side effects or distrusted the vaccine (or both) for diverse reasons (Table 3): its composition, insufficient tests, or general concerns dating back to the mass vaccination campaign for pandemic influenza in 2009, or alleged risks of other vaccines (e.g. against hepatitis B/multiple sclerosis, Table 3, P6). Finally, some displayed complacency or procrastination (Table 3, P15).

### Trust in medicine, science, pharmaceutical industry, authorities

Most vaccinated and unvaccinated patients reported trusting their physician — primary care practitioner or specialist — for the management of their disease (Table 4). All vaccinated patients trusted the SIV except one (P13), who had doubts about its efficacy but continued to be vaccinated and grew suspicious of SIV during the 2009 pandemic (Table 4). Nonetheless, some vaccinated patients reported a more or less pronounced mistrust of the risks associated with drugs generally — an attitude they shared with the majority of unvaccinated patients — and/or a turn toward alternative medicine (Table 4, P13).

*“No I don’t take anything, it’ll go away, and so I am opposed to any drug, whether it’s homeopathy or conventional...”* (P10)*.*

Several unvaccinated participants reported trusting both their doctors and scientists who develop vaccines, thus trusting vaccination in general (Table 4). But they shared with most of the other unvaccinated patients mistrust in pharmaceutical companies and/or public health authorities. The following statement by Patient 10 summarises a typical line of reasoning to justify the perception that pharmaceutical companies do insufficient testing and pay inadequate attention to safety when they produce vaccines:

*“I tell myself that the vaccines caused it...but maybe I’m wrong... they developed them a little rapidly, perhaps with a profit motive behind it, because I think it must be a cutthroat competition to be the first drug company to have a vaccine”* (P10)*.*

Several unvaccinated patients reported they were deeply concerned about how the public health authorities had conducted the vaccination campaign against the A/H1N1 pandemic in France in 2009; they considered it a “fiasco” and questioned its utility (Table 4, P19) and, beyond that episode, the utility of systematic vaccination. Several patients reported greater mistrust of SIV since 2009.

**Table 4:** Results of the thematic analysis according to the trust dimension^a^: vaccinated and unvaccinated patients with diabetes

Category	*n*	*Examples from the interview transcript*s
Vaccinated patients (*N* = 8)		
Trust in one’s physician -- specialist or general practitioner (request for advice, following their recommendations)	5	*I talked about with her (my general practitioner), and she told me that it’s in my best interest, so I said, ok, I’ll do it, and then...* (P9)
Suspicion of drugs generally and/or recourse to alternative medicine	2	*I know you should not use too much of it* [conventional medicine]*, when he gives me medication to take for 3 weeks or a month, I tend to stop earlier … because I think that this kind of medicine also destroys one’s health a little* (P13).
Trust in the composition of vaccines	2	*…they are drugs...it’s vaccines × years and so I think that there would have been many many more problems, no, no I’m confident...* (P18).
Suspicion of influenza vaccine at the time of the pandemic	1	*There are times when there was a history of vaccination for the bird flu, there...it was a little bizarre this period then, so ... I didn’t do it ... and why? I don’t know, because they said so many things about it that, well* (P13).
Unvaccinated patients (*N* = 11)		
Trust in one’s physician -- specialist or general practitioner	7	*...From the moment that the doctor told me, I’m giving you this medicine, it’s for this ... [...] so I trust the doctor...* (P3). *If I had a problem, I would ask my doctor; she would give me a clear answer and then if it was serious, she would tell me or give me an alternative...* (P14).
Trust in vaccines because of trust in science to develop them	3	*Fortunately vaccines exist for all diseases...it’s a good thing that the vaccines that researchers concoct for us protect us from all the crap that floats around!* (P15).
Suspicion about drugs and/or recourse to alternative medicine	6	*No I don’t take anything, it’ll go away, and so I am opposed to any drug, whether it’s homeopathy or conventional...* (P10). *I even look at other medicines, a lot at Chinese medicine... A lot of phytotherapy, a lot with essential oils... that is, in diabetics, as soon as they have a fever ... an antibiotic is prescribed... so I compensate for that with essential oils...* (P3).
Suspicion of vaccines because of mistrust of the pharmaceutical industry and/or French public health authorities	8	[Composition of vaccines] *we don’t know what they put in them, we don’t know* (P3). *And then there was that thing with H1N1, the ultimate, so I said no, no... but even before that I was doing it already because I didn’t perceive the utility, the need, and then after, it encouraged me in a... in the analysis that I’d done before…* (P10).

^a^Trust in one’s doctor, in medicine, science, pharmaceutical industry, and authorities

## Discussion

### SIV: stable habits in a stable environment

Our study of patients with diabetes found that SIV-related behaviours are mainly stable, dictated by habit, as previously shown in the general population [7]. In the prevention field, *past behaviour* is usually predictive of subsequent behaviour [26], probably because when a behaviour is carried out *regularly* in a *stable context*, responses are performed fairly automatically, without either conscious decision-making or thinking [27]. In France, the health insurance fund sends coupons for free SIV each year to people in the at-risk groups. This procedure makes SIV accessible and also serves as a routine reminder, as do regular visits to physicians monitoring the diabetes and recommending SIV. Trust in these doctors contributes to a stable environment promoting the habit of vaccination, once the behaviour has been adopted [9, 13, 19, 27–29]. Inversely, we found that this environment had little influence when the patient decided against SIV suggesting that this behaviour is hard to modify.

### Perceptions anchored in past experiences, persistence of “false beliefs”

As already observed [13, 30, 31], patients’ perceptions and decisions about vaccination were often anchored in their own or their family and friends’ past experience. Living through the disease can increase self-perceived vulnerability and promote the adoption of behaviour intended to prevent it [15, 32]. Patients may be more likely to develop the habit of vaccination if their initial experience with SIV is positive [9]. The experience of an influenza-like illness (ILI) after SIV, however, raised questions about its effectiveness in patients’ eyes. Those past experiences were sometimes interpreted in accordance with patients’ “false beliefs”: believing that SIV protects against colds, or that it inoculates them against the disease. Influenza is rarely diagnosed by virological testing (PCR) so that patients (and doctors) often cannot distinguish between true influenza, influenza-like illness, or a general post-vaccination reaction. This uncertainty explains and maintains these misconceptions, which physicians must attempt to explain to patients. They might also usefully use virological testing more systematically.

### Influenza trivialisation & compensatory health beliefs: strong barriers to SIV

Contrary to findings about the 2009 pandemic [28], collective protection (of one’s family or others) was rarely reported as a driver for SIV uptake and fear of severe side effects was not the most frequent reason for refusing SIV. Unvaccinated patients justified their choice by a range of attitudes — denial, trivialisation, or relativisation of the risks (Table 3) — that have also been observed for SIV [13] and other types of behaviour (e.g., smoking) [33–35]. Familiarity with vaccine-preventable-diseases often decreases vaccine acceptance [36]. Beliefs that influenza can be controlled by avoiding exposure or by curative treatment can be interpreted as a perceived control over risks [37]. They are also called “compensatory health beliefs”, that is the belief that the negative consequences of an unhealthy behaviour can be compensated for or neutralised by engaging in another health-protective behaviour. Compensatory health beliefs are assumed to operate as a justification preceding, accompanying, or following the rejection of a health behaviour (here SIV) [13, 27, 37] strengthening the conviction this is the right choice.

### Some discordances between behaviours and perceptions

The perceptions of risks were often different between vaccinated and unvaccinated patients in accordance with HBM hypotheses for SIV. However, the adoption of behaviours may sometimes induce a revision of perceptions, to avoid cognitive dissonance [30]. Discordances sometimes existed between perceptions and behaviour: remaining vaccinated despite the feeling that SIV lacks efficiency; remaining unvaccinated despite feelings of vulnerability towards influenza complication. Such discordances might be a marker of hesitancy (to maintain SIV or adopt it as patients’ citations suggest) and suggest a behaviour inertia, that is, the persistence of behaviour (or non-behaviour) despite perceptions contrary to it. In such situations, advice from a trusted physician is important to convince the patient to adopt SIV or dissuade her/him quitting SIV.

The large majority of unvaccinated patients with diabetes trusted their physicians, contrary to previous observations among adults regarding SIV [19]. The transcripts suggest that trust of physicians is built in a close, sustained, and concrete doctor-patient relationship to monitor/treat the diabetes. But distrust in distal stakeholders — the French government and pharmaceutical companies — was more marked in unvaccinated than vaccinated patients. Management of the 2009 A/H1N1 pandemic, by what the public perceived as exaggeration of the risks and minimisation of the agency of the population, probably impaired the credibility of those stakeholders, including for the information they disseminate about SIV [12, 38]. SIV uptake decreased steeply after the 2009 A/H1N1 pandemic and has continued to drop since then [6]. Nonetheless, our results show the presence of distrust in allopathic (conventional) medicine in general especially but not exclusively among unvaccinated patients. Several public health controversies during the last decades in France (e.g., the 2009–2010 drug safety scandal (benfluorex, Mediator^®^) [39] and the late reaction of health authorities to them might have fostered a climate of more widespread public distrust.

### Limitations

The results of this study must be interpreted prudently in view of the limited number of interviews. This number nonetheless enabled thematic saturation. Moreover, the sample was diverse for several social and demographic variables as well as for types of diabetes (Table 1). For 15 of the 19 participants, the time since onset of their diabetes limited the exploration of the decision-making process at the moment that they chose or rejected SIV. But including only people newly diagnosed with diabetes would not have allowed us to achieve the first objective of this study and presented questions of feasibility.

## Conclusions

This study confirms previous published findings showing that beliefs and perceptions about influenza and SIV influence the SIV behaviours of patients with diabetes. Our results underline that SIV rejection may be durable and influenced little if at all by policies facilitating access to SIV (such as vouchers). They thus suggest the importance of recommending SIV at every opportunity and of interventions to increase community demand for SIV. Some interventions have proved effective in these situations (e.g., telephone calls from other older peers) [40]. Patients’ frequent false beliefs and compensatory health beliefs relative to SIV call for better health education to patients by healthcare professionals, who in turn should be better trained to provide it. Virological testing to diagnose severe ILI to limit misunderstanding about SIV effectiveness might also be considered. Finally, restoring trust in health authorities and developing new vaccines with improved clinical effectiveness [10, 21] are also necessary.

## Appendix 1

### Interview guide

- **Your health:**
  - **Summary of your diabetes:**
    - What type of diabetes? Diseased screened recently or quite a while ago? Disease stage? Complications: yes or no?
  - **Diabetes management:**
    - Do you belong to an association of people with diabetes?
    - How did you learn to handle your insulin to manage and control your diabetes? To prevent and handle hypoglycemia? To handle meal composition?
- **Your physician:**
  - **Medical monitoring:** Regular diabetes monitoring? Yes/No, Why?
  - **Nature of the support/monitoring:** From a primary care physician? Specialists? A hospital?
  - *Discuss first relationships with primary care physicians and then with specialists:*
    - How did you choose him/her? Have you changed doctors? Why?
    - How do you get along with your doctor? What do you expect of him/her?
    - Do you ask him/her the questions you want during visits?
  - **Hospital consultations:** Frequency of visits? The reasons you see a doctor at a hospital?
  - **Trust in the medical field:** Quality of relationships with physicians, use of alternative medicine?
  - Do you look for additional information beyond what your doctor tells you? What are your sources?
  - Do you discuss vaccinations with your physician?
- **Your vaccinations:**
  - **Patients with diabetes: particular at-risk population for whom vaccination is recommended**
  - Have you been advised of the vaccines recommended for you?
    - *Influenza*
    - *Pneumococci*
  - Informed by the general practitioner? Or by the health insurance fund? Other
  - **Experience of disease complications, link with vaccination.**
  - **Has your doctor recommended that you be vaccinated?**
  - If so, what is the last vaccine that s/he recommended or administered?
  - Did the vaccination take place at the time it was recommended? Or was it delayed? Why?
  - Did you hesitate? Tell me how that went?
  - **Did you ask yourself some questions before making your decision?**
  - On the topic of vaccines, these questions might have included:
  - **Diseases that the vaccine protects against:** Do you know the diseases it protects against? Were you able to discuss the diseases it protects against with your physician?
  - **Indications and contraindications of a vaccine:** Did you read the labelling leaflet that came with the vaccine? What did your doctor say about the vaccine’s indications and contraindications?
  - **Side effects of a vaccine**: Did you read the labelling leaflet that came with the vaccine? Do you have any doubts about the vaccine’s composition? What do you think are its side effects? Were you able to discuss the question of possible side effects with your doctor?
  - If the person mentions side effects:
  - What made you think about the side effects?
  - Was it something in particular? In the composition of vaccines?
  - How did you come to be interested in these components?
- **Your sources of information about health in general and vaccination in particular:**
  - When you need information about a problem in particular about a question that worries you about your health or more. Particularly about your diabetes: what do you do?
  - Do you talk to your doctor?
  - Do you have sources of information other than your doctor?
  - Which? (spouse, family, friends, relation, media/news, internet...)
  - *Depending on the response about sources, ask the person about the sources they did not spontaneously mention, why: source deliberately not considered or ignored?*
  - Do you have personal examples or examples from your family, friends, or relatives of experiences endured or reported in relation to vaccination?

## Abbreviations

HBM: Health belief model
SIV: Seasonal influenza vaccination
